# Confronting situations of violence against children and adolescents from the perspective of Guardianship Counselors

**DOI:** 10.1590/1980-220X-REEUSP-2022-0322en

**Published:** 2022-11-28

**Authors:** Aline Cammarano Ribeiro, Fernanda Ilha Pedroso, Jaqueline Arboit, Fernanda Honnef, Cristiane Cardoso de Paula, Tifany Colomé Leal, Maria Helena Cunha Brum

**Affiliations:** 1 Universidade Federal de Santa Maria, Santa Maria, RS, Brazil.; 2 Universidade do Estado de Santa Catarina, Chapecó, SC, Brazil.

**Keywords:** Violence, Child, Adolescent, Child Advocacy, Qualitative Research, Violencia, Niño, Adolescente, Defensa del Niño, Investigación Cualitativa, Violência, Criança, Adolescente, Defesa da criança e do Adolescente, Pesquisa Qualitativa

## Abstract

**Objective:**

To describe the potentialities and limits in confronting situations of violence against children and adolescents seen from the perspective of Guardianship Counselors.

**Method:**

Qualitative research, developed with 18 Guardianship Counselors from two municipalities in the central region of the State of Rio Grande do Sul, Brazil. Data were generated between June and July 2021, using the focus group technique, and were subjected to thematic content analysis.

**Results:**

The following were identified as potentialities for combating violence against children and adolescents: denunciations; networking; and media coverage. As limits, we have: naturalization of violence; pandemic of the Covid-19; de-structuring of the intersectoral network; lack of training; and difficulty in developing preventive actions.

**Conclusion:**

The Guardianship Councilors require training to act in cases of violence against children and adolescents, as well as support from other sectors, such as health, education, public safety, and social assistance, in order to fully attend to these cases.

## RESUMO

**Objetivo:**

Descrever as potencialidades e limites no enfrentamento das situações de violência contra crianças e adolescentes na perspectiva de Conselheiros Tutelares.

**Método:**

Pesquisa qualitativa, desenvolvida com 18 Conselheiros Tutelares de dois municípios da região central do Estado do Rio Grande do Sul, Brasil. Os dados foram gerados entre junho e julho de 2021, por meio da técnica de grupo focal, sendo submetidos a análise de conteúdo temática.

**Resultados:**

Elencaram-se como potencialidades para o enfrentamento da violência contra crianças e adolescentes: denúncias; atuação em rede; e divulgação midiática. Como limites, tem-se: naturalização da violência; pandemia da Covid-19; desestruturação da rede intersetorial; ausência de capacitações; e dificuldade para desenvolver ações de prevenção.

**Conclusão:**

Os Conselheiros Tutelares requerem instrumentalização para atuar nos casos de violência contra crianças e adolescentes, bem como o apoio dos demais setores, como saúde, educação, segurança pública e assistência social, de modo a atender integralmente esses casos.

## RESUMEN

**Objetivo:**

Describir las potencialidades y los límites en el enfrentamiento de situaciones de violencia contra niños y adolescentes vistos desde la perspectiva de los Consejeros Tutelares.

**Método:**

Investigación cualitativa, desarrollada con 18 Consejeros Tutelares de dos municipios de la región central del Estado de Rio Grande do Sul, Brasil. Los datos fueron generados entre junio y julio de 2021, utilizando la técnica de grupos focales, y fueron sometidos a análisis de contenido temático.

**Resultados:**

Se identificaron como potencialidades para el combate a la violencia contra niños y adolescentes: las denuncias; el trabajo en red; y la cobertura mediática. Como límites, tenemos: naturalización de la violencia; pandemia del Covid-19; desestructuración de la red intersectorial; falta de capacitación; y dificultad para desarrollar acciones preventivas.

**Conclusión:**

Los Consejeros Tutelares requieren capacitación para actuar en casos de violencia contra niños y adolescentes, así como el apoyo de otros sectores, como salud, educación, seguridad pública y asistencia social, para atender plenamente estos casos.

## INTRODUCTION

Violence against children and adolescents constitutes a global public health problem and a violation of human rights of this population. It is characterized by any action or omission perpetrated by parents, relatives, legal guardians, institutions and society, resulting in physical, emotional, sexual and moral harm^(1)^.

A systematic review of population surveys estimated that, worldwide, up to 1 billion children aged 2 to 17 years had suffered some form of violence in the previous year^(2)^. Among these, national and international literatures point to physical violence^(3–6)^; emotional/psychological violence^(3, 4, 6)^; sexual violence^(3, 4, 6, 7)^; and neglect^(3, 4, 6)^.

Exposure to violence in the population of children and adolescents can occur at home, at the community/public, or at the school setting^(5, 6, 8)^. The impacts of this violence are diverse, causing individual, family, social and economic losses. From this perspective, the main risk is the one for the development of mental disorders, such as post-traumatic stress disorder, emotional and behavioral problems, and symptoms of depression^(8)^. The cognitive functions, health and well-being^(3)^, sleep^(4)^, and school performance^(9)^ of children and adolescents who experience violence are also affected by the problem. Furthermore, there is evidence that children and adolescents have contracted HIV through unprotected sexual contact due to sexual violence in childhood^(10)^.

In Brazil, the first movements geared towards guaranteeing rights of children and adolescents came with the Federal Constitution of 1988. By means of article 227, it states that it is the duty of the family, the society, and the State to guarantee to children and adolescents, among others, the right to life, health, food, education, leisure, dignity, respect, and freedom, protecting them from all forms of neglect, discrimination, exploitation, violence, cruelty, and oppression^(11)^.

These rights were recognized in 1990 by Law No. 8069/90, which provides for the creation of the Statute of the Child and Adolescent and other provisions. In its article 5, the Law established the Guardianship Council, a permanent and autonomous body, present in each Brazilian municipality and administrative region of the Federal District^(12)^. The Guardianship Council integrates the care and protection network for the comprehensive care of children and adolescents in situations of violence. In order for its actions to be resolutive, it must develop them in an integrated way with other services and sectors, such as health, education, social assistance, public security, and the judiciary^(1)^.

In view of the above it is highlighted that, although violence against children and adolescents is object of different investigations in the health area, the productions are scarce when the focus is on the actions of Guardianship Counselors, pointing out a gap of investigations that unveil the perspectives of these professionals, therefore justifying this study. Moreover, this research meets the 2030 Agenda for Sustainable Development, which recognizes the need to eliminate all forms of violence. For these reasons, this study seeks to answer the following guiding question: What are the potentialities and limits in dealing with situations of violence against children and adolescents from the perspective of Guardianship Counselors? Therefore, it aims to describe the potentialities and limits in dealing with situations of violence against children and adolescents from the perspective of Guardianship Counselors.

## METHOD

### Study Type

Exploratory-descriptive study, using a qualitative approach. The Consolidated Criteria for Reporting Qualitative Research (COREQ) were applied to ensure the quality and transparency of the writing.

### Research Setting

The study was developed in the Guardianship Councils of two municipalities in the central region of the State of Rio Grande do Sul, Brazil.

### Population and Selection Criteria

In the municipalities that comprised the study setting, there were four Guardianship Council teams, three in one of the municipalities and one team in the other. These teams totaled 20 Guardianship Councilors, who were invited to participate in the study, making up a convenience sample. Despite the acceptance of all the Councilors, two of them were unable to participate on the dates established for the generation of the data, due to emergency care.

The inclusion criteria were: to be a Guardianship Counselor of the municipality and to have been in office for more than six months. The exclusion criteria included being on vacation, on leave of any kind during the data collection period, or unavailable on the data collection date due to demands of the service itself.

### Data Collection

The focus group technique was used to generate the data. This is a qualitative research technique based on group interactions that aim to collect information about a specific theme from a group of selected participants^(13)^.

It is noteworthy to point out that, prior to data collection, the coordinators of each Guardianship Council team were contacted by telephone to present the proposal and invite the Councilors to participate in the study. At this moment, agreements were made as to the dates and times of the group sessions.

In order to meet the objective proposed by the study, two focus group sessions were developed with each team of Guardianship Counselors, totaling eight group sessions. Three to five Guardianship Counselors participated in each session, according to the availability of the participants. The group sessions were guided by a script, which aimed to subsidize the collection team as to the execution of the meeting and discussions. The script covered topics related to the Guardianship Counselors’ perspectives on strategies for dealing with violence against children and adolescents.

The data collection took place between June and July 2021, face-to-face, in a large room reserved in the headquarters of each Guardianship Council team. The protocols for preventing COVID-19 according to each municipality and its health care services were considered.

The sessions were organized along the following moments: opening and group contract, discussion, validation and synthesis of information, and closing. They lasted an average of one hour and thirty minutes. The research team was composed of the responsible researcher (moderator) and a research assistant (observer). The moderator was experienced in the development of the focus group technique and coordinated the group sessions so that they were conducted in an atmosphere of empathy and flexibility, in which the participants could talk about their perspectives in relation to the phenomenon. The observer tried to capture the participants’ non-verbal reactions/information, recording it in a field journal. The group sessions were recorded using a digital recorder (audio) aiming at a reliable data transcription. The closure of the fieldwork was determined when the internal logic of the object of study was understood^(14)^.

### Data Analysis and Treatment

To systematize the data, first the data from the focus group sessions were transcribed verbatim using the online program *oTranscribe*. After that, the content was directed to a text editor program, constituting the research corpus. Subsequently, it was submitted to thematic content analysis, composed of three phases: pre-analysis, material exploration, and treatment of the results obtained and interpretation^(15)^. In the pre-analysis, the recordings were listened performing floating reading to reveal the initial impressions about the material. After, through exhaustive readings and application of the chromatic technique, the excerpts of the participants’ speeches were highlighted, constituting material to be subjected to further analysis. In the material exploration phase, the common information found in the content of the transcribed speeches was cut out, subsidizing the constitution of two thematic categories: P*otentialities for confronting violence against children and adolescents*; and *Limits for confronting violence against children and adolescents*. In the phase of treatment of the results obtained and interpretation, inferences were proposed based on the objective of the study. Thus, the data were analyzed according to the theoretical foundation relevant to the theme.

### Ethical Aspects

The study is in compliance with the recommendations expressed in Resolution No. 466/2012 of the National Health Council, which provides for the development of research with human beings. Thus, data collection was initiated after approval by the Research Ethics Committee of the Federal University of Santa Maria, under Approval Opinion No. 4,671,367, in the year 2021. Furthermore, the study participants were informed about the objectives, method, risks, and benefits of the research by reading and explaining the Informed Consent Form. Those who agreed to participate in the study signed two copies of the consent form, one of which was given to the participant and the other to the researcher in charge. To ensure anonymity, the testimonies will be identified by the abbreviation CT, referring to Guardianship Counselor, followed by the number corresponding to their order of participation in the study (e.g.: CT1, CT2, CT3… CT18).

## RESULTS

Eighteen Guardianship Counselors participated in the research. Regarding their characterization, the majority were female (n = 16). Their age ranged from 31 to 63 years, with a mean of 46.9 years. As for color, the majority declared themselves to be white (n = 11). Regarding the level of education, the majority of the participants had completed higher education (n = 14). The time they have worked for the Guardianship Council varied from 1 to 10 years. Regarding professional experience, six participants reported having already worked in other organs of assistance to children and youngsters.

### Potentialities to Confront Violence Against Children and Adolescents

In this category, the Guardianship Counselors mentioned aspects that enhanced their ability to confront situations of violence perpetrated against children and adolescents. They are: denunciations made by the victims or third parties; networking, especially between the actors in the health and education sectors; and the media dissemination of the possible signs for the identification of violence and the importance of making denunciations.

Regarding the reports, the participants revealed that it is one of the main ways they have to identify the violence experienced by children and adolescents. These reports come from the victims themselves or, even, from the family’s neighbors during the counselors’ visits.

*I believe* (as a potentiality) *in the spontaneity of the child or adolescent who suffers* (violence) *coming to talk.* (CT1)

*A very important factor is that when we go for a visit, the neighbors come to us and tell us what happens. So, in fact, this is a very strong factor, the spontaneity of the neighbors or the victim herself in making the complaint*. (CT10)

The participants also mentioned networking as a potentiality for dealing with violence. In this context, they highlighted the education sector through schools, and the health sector, through the Family Health Strategy (FHS) and professionals such as nurses and Community Health Agents (CHA).

In schools, the bond promoted by the daily contact with teachers allows them to suspect that children or adolescents are suffering some kind of violence, because they present physical marks or some behavior that is different from the usual. The health professionals of the FHS, due to the fact that they develop their activities in the territory where these children and adolescents in situations of violence live, can suspect such situations and communicate their suspicions to the counselors.

*I think that one of the factors that facilitates a lot and has harmed us now* (by the pandemic) *is the schools (…) in the schools the children go every day and they can notice any spot on the child, if she gets depressed (…) there are many people’s eyes there* (referring to teachers). (CT3)

*It’s the partners like the school, the teachers that identify this kind of problem a lot* (referring to violence against children and adolescents). (CT6)

*The school is a very important factor because we receive the denunciation, we call the school and ask about the student, to know if they noticed if the child or adolescent is different, if they arrive with marks* (…) (CT18)

*A health network in the territory, with Community Health Agents that can always be giving attention to the population. I think that with these network partners, it is much easier to confront violence against children and adolescents*. (CT6)

*Also the network; when we refer to the ESF (…) sometimes the nurse herself or the Community Health Agent comes and contacts us. When they ask to perform some exams, when the vaccines are sometimes late (…) we realize that that mother is neglecting us in some way.* (CT12)

The media, through the press as well as social media such as Facebook, has contributed to the fight against violence against children and adolescents. Through these communication channels, materials such as videos are disseminated, helping society and the victims themselves to identify signs of violence and the need to seek help. Furthermore, by disseminating information about the relevance of reporting and the organs to be sought, such as the Guardianship Council, the media encourages these reports to be made.

*Today, one thing that has helped us is the work done in the press. This encourages people to denounce, to look for us, which was not encouraged in the past. I don’t know if you have seen, on Facebook, videos of signs that can be used for us to identify that a child or an adolescent is in a situation of violence or for them to identify themselves that they are being abused, that they are in need of help. These things also make it easier for people to gain confidence in making denunciations.* (CT2)

*One of our interventions was on the university radio. There we broadcasted the work of the Council of Guardianship that reached out to the families and many people who saw that there was a problem sought out the Council. So, the radio has a lot of power and reaches a lot of people* (CT3)

### Limits to Confronting Violence against Children and Adolescents

This category presents the factors that limit the confrontation of situations of violence against children and adolescents, which are: naturalization of violence; the Covid-19 pandemic; disorganization of the intersectoral network; lack of training; and difficulty in developing preventive actions.

The Guardianship Counselors reported the naturalization of violence, especially physical violence, as a limitation in dealing with violence. They associated this naturalization with cultural issues, such as education, which end up leading the seriousness of the situation of violence to be disregarded.

*One of the things is the cultural aspect (…) “ah, I was abused, assaulted, I’m here”, you know? They don’t take it seriously, it’s not as if it was an aggression. It’s as if it was normal, as if the situation wasn’t serious.* (CT4)

*It is impressive how they still use physical violence to educate* (CT1)

The Covid-19 pandemic was also mentioned as a limiting factor by one of the participants. For this participant, the context of restricted family life and non-attendance at school has been detrimental to the identification and confrontation of situations of violence in the population of children and adolescents.

*Right now, I think one of the limiting factors is the pandemic itself, because we had a reclusion of children and adolescents within their families and we lost the reference that we had as a school, in observation.* (CT6)

The participants also mentioned as one of the limits the lack of structure of the intersectoral network of care to children and adolescents. In this sense, they cited difficulties in communication and in making referrals, the discontinuity in the services, the high demand, and the deficit of professionals in the services that make up the network, which leads to the slowness and non-performance of the required services.

*The limitation I think is a matter of the functioning of the network itself, which is not shared as it should be for a more agile action. There is a lack, sometimes, of professionals and communication* (CT3)

*The lack of support in the sense of counter-referrals, of the network, of referrals, this limits us (…) when we refer to CRAS (Reference Center for Social Assistance), CREAS (Specialized Reference Center for Social Assistance), the team is out of capacity*. (CT5)

*The lack of continuity (in the care of the child or adolescent), because we request the service, make the referrals and then stop. So, this limits us a lot in giving continuity in that service*. (CT9)

*The lack of attention from the network is also a limit. Sometimes we forward them, but it takes a long time for them to be attended (…) it is very slow*. (CT8)

*Our demand here is very high (…) to keep following up is what makes it difficult sometimes for us, because here many complaints arrive, every day.* (CT12)

*In my point of view, I think that there is a lot of demand and few professionals. We have, for the violence part, only one psychologist to serve our entire city. She only takes away the suffering, that momentary pain, but that service that should last a year or more, she can’t do it.* (CT14)

The Guardianship Counselors reported the lack of training to act in the confrontation of violence against the population of children and adolescents. The fact that they do not have this training limits, among others, the performance of different actions to prevent violence, such as lectures.

*This year, for example, we didn’t take any courses. And, in fact, we should be always being trained, and this doesn’t happen. There is little investment in our area.* (CT13)

*I participated in the other administration, but the colleagues* (Tutelary Counselors*) who took office now haven’t participated in any training yet. If they* (colleagues) *didn’t have the interest to study and research the Statute, we would have nothing (…)* (CT6)

*The Statute for Children and Adolescents (ECA)* (referring to this statute) *says that we need to do preventive actions together with the protection network. But I can’t give a lecture if I am not trained to do so* (CT1)

Another limit reported in dealing with situations of violence is in the development of preventive actions. The participants mentioned that the actions of the Guardianship Counselors are usually taken when there is already a suspicion or a complaint, hardly ever developing preventive actions.

*We can hardly prevent anything. When the Council receives a complaint or a suspicion (…) the problem is already there. Unfortunately, that’s how it is. The violence is already installed.* (CT9)

*We should also work with the issue of prevention, but what we do is to put out fires. We are more guaranteeing rights than preventing things from happening* (CT13).

## DISCUSSION

The analysis of the data coming from the Guardianship Counselors pointed out potentialities and limits in the confrontation of situations of violence against children and adolescents. Regarding reports, the victims’ spontaneous reports and the neighbors’ reports were pointed out by another research as aspects that contribute to the identification of situations of violence^(16)^. However, the denunciation by the children themselves in situations of violence is limited, especially if this situation affects the age group of younger children who cannot verbalize about their victimization, worsening when the perpetrators are family members or caregivers^(17)^.

In the context of the confrontation network, the actions of the health sector through the Family Health Strategy (FHS), and professionals such as nurses and CHAs were pointed out. The FHS, due to the proximity to families in its coverage territory, is the main gateway to the Unified Health System (SUS) and its multidisciplinary teams, through home visits, for example, have the potential to access families in situations of greater social vulnerability, and can play a key role in the identification of violence against children and adolescents, notification and referral of cases^(18)^.

Regarding the performance of nurses, a study that explored the experiences of these professionals, in providing care to victims of child violence, revealed that this care corresponded to four main categories of roles, namely: protection, diagnosis of violence, notification and education. However, they highlighted the limited teaching that they receive regarding the topic, since at no time during their training had they received guidance on providing care to victims of child violence^(19)^.

The role of the CHAs is also highlighted by their insertion both in the health care network and in the community where they work. This is an important actor in the articulation of actions within the network, because the link with the population and the opportunities provided by regular home visits to families are possibilities to identify situations of violence and subsequently trigger the Council of Guardianship^(20)^.

Despite the relevant role of health professionals facing the problem, the health sector is unprepared to act in situations of violence against children and adolescents, which restricts its actions to biomedical care, focused on treatment and medicalization, especially when there are apparent injuries^(21)^. Acting focused on biological issues may be related to a series of weaknesses originated during training, which generates a deficit of knowledge and skills that contributes to the fact that professionals are faced with the problem and are not able to provide adequate and comprehensive care. Based on this, the relevance of professional training and in-service education programs for professionals in the sector is pointed out, addressing, among other aspects, the identification of situations of violence, the prevention and confrontation actions of such situations, based on the perspective of comprehensiveness.

The school was also pointed out as a constituent of this confrontation network. As it is a daily living environment, and often the only one outside the family circle, to be in the school make it possible for teachers to detect signs and symptoms of violence, including changes in children and adolescents’ behavior or apparent injuries^(21)^. Thus, the proximity, systematic contact and daily living of this group with educators makes them in a strategic position for the identification of this harm^(9, 18, 22)^. Additionally, it is possible to carry out violence prevention actions with children and adolescents at school, as it is a privileged environment for these actions^(9, 18)^. As an example of these measures, the creation of welcoming and listening spaces for students and the implementation of educational activities are cited^(9)^.

To integrate the family to the actions developed at school, seeking to strengthen the family-school bonds, is essential for the confrontation of violence in the child-youth population, since it can contribute to understanding the behavior of students and possible situations of vulnerability that they may be experiencing, such as domestic abuse^(9)^. In this context, there is the possibility of promoting the (re)construction of affection relationships in the family nuclei, being a path to overcome its violent history^(23)^.

The initiatives to disseminate the problem of violence against children and adolescents mentioned by the Guardianship Counselors are intended to provide subsidies for the recognition of a situation of violence. Thus, it is evident that the greater knowledge on this issue encourages the complaints for its identification, both by the victims, their families, and the society^(24)^. A study aimed to assess the knowledge, motivation and self-reported communication of parents to their children about personal safety and prevention of childhood sexual abuse, pointed out that, after parents watched a video on the subject, they were motivated to talk to their children about violence^(25)^. This reveals the potential of this type of tool in the dissemination of information, for prevention and confrontation of violence against this population.

In regards to the limiting factors for confronting violence against children and adolescents, the influence of cultural elements emerged, such as physical violence used as an educational practice. It is evident that corporal punishment continues to be accepted and employed by parents as a method of discipline^(26)^. In view of this, it is necessary to develop actions with parents and caregivers, seeking the denaturalization of violent practices as an educating mechanism, which is widespread in society. Seeking to strengthen the bond and dialogue in the family nucleus is one of the pillars for a healthy family relationship without violence.

Despite scarcely emphasized in the reports, the Covid-19 pandemic was also considered a factor that limits the confrontation of violence against children and adolescents, especially regarding social distancing and the loss of school attendance. Among the impacts generated by the pandemic, there is the greater permanence in the family environment, where most violent acts against this public are perpetrated^(27)^, as well as the physical and affective distance from the school environment^(22)^. The permanence at home and the impossibility of living with a reference adult also hinder the search for help in these cases^(28)^. In the same direction, research points out an increase in the actual number of cases of child violence in the pandemic period, while the social distancing, with the closing of schools and other services, had as a consequence the limitation of attention and care to children and adolescents in the period^(18)^, creating obstacles for the identification of cases and facing the problem.

Regarding the integration of the protection network, the reports of the Guardianship Councilors show that the institutions and employees have not shared their responsibilities, acting in isolation. From this perspective, the absence of an interconnected network leads to the recurrence of situations of violence against children and adolescents, creating difficulties for their identification and confrontation due to the lack of continuity of the assistance requested by the Guardianship Council. For this reason it is urgent the active performance of the actors of this network, aiming at resolutive actions and breaking the cycle of violence^(29)^.

The actions to prevent violence are limited due to the lack of technical skills, training and high demand. From this perspective, there is divergence between the reality of the service and what is recommended by the ECA, which establishes the implementation of preventive actions with the community and protection network teams, for the recognition of symptoms of possible violence against children and adolescents^(12)^.

Following this line of reasoning, in order to protect this population, it is essential that these actors are trained according to the current literature, in order to develop preventive actions. Faced with this need, programs like PREVENT (Preventing Violence through Education, Networking, and Technical Assistance) have emerged. This program offers educational interventions based on pre-established and free pedagogical guides, and programs offered by governmental entities that present, among others, teaching methodology and content for professional training, focused on education and technical assistance based on evidence for the prevention of violence^(30)^.

The limitation of the study is that it was carried out with Guardianship Counselors from only two municipalities in a single region of Brazil, characterizing a study at local level, that may not reflect the reality of other territories, which makes it impossible to generalize the results.

This study provides visibility to violence against children and adolescents in the voice of Guardianship Counselors. The complexity and multi-causality of violence point to the need to develop interdisciplinary and intersectoral approaches and interventions for the prevention and confrontation of this problem. Thus, it will be possible to promote the rights of children and adolescents to grow and develop in safe environments, free from any form of violence.

## CONCLUSION

Among the potentialities to confront violence perpetrated against children and adolescents are the reports made by the victims themselves or by neighbors; the networking of the health sector represented by the FHS, especially by nurses and CHAs, and of the education sector represented by schools and teachers; and also the media dissemination in the press and social media about information for the identification of violence and the relevance of reports. On the other hand, the naturalization of violence, especially physical violence used as an educational practice; the pandemic of COVID-19, due to the social distancing and lack of contact with the school; the lack of structure of the intersectoral network; the lack of training of Guardianship Counselors to act against violence; and the difficulty of actions to prevent violence against children and adolescents are limits to the confrontation of violence in the infant-youth population.

Despite the potentialities pointed out by the Guardianship Counselors, these professionals require training to act in cases of violence against children and adolescents. In addition, it is urgent that the Guardianship Councils rely on the support of the intersectoral network, such as health, education, public safety, and social assistance, in order to fully assist these cases. Thus, it is recommended that intervention studies be carried out aiming at changes in the assistance practices, such as the establishment of assistance flows in the municipalities.

## DATA AVAILABILITY

https://doi.org/10.48331/scielodata.WMQITQ
